# Comparative study comparing endoscopic thyroidectomy using the axillary approach and open thyroidectomy for papillary thyroid microcarcinoma

**DOI:** 10.1186/1477-7819-10-269

**Published:** 2012-12-12

**Authors:** Hayemin Lee, Jina Lee, Ki Young Sung

**Affiliations:** 1 Department of Surgery, Bucheon St. Mary’s Hospital, The Catholic University of Korea, Sosa-dong, Wonmi-Gu, Bucheon City, Kyunggi-Do, 420-717, Korea

**Keywords:** Endoscopic thyroidectomy, Conventional open thyroidectomy, Micropapillary thyroid carcinoma

## Abstract

**Background:**

Endoscopic thyroidectomy has been applied prudently to malignant thyroid tumors. The purpose of our study was to compare the surgical outcomes of endoscopic thyroidectomy (ET) and conventional open thyroidectomy (COT) for micropapillary thyroid carcinoma.

**Methods:**

From October 2002 to December 2008, 78 patients underwent unilateral lobectomy and isthmectomy with central lymph node dissection for papillary thyroid microcarcinoma. Of these, 37 patients underwent ET and 41patients COT. Surgical outcomes, including operation time, number of retrieved lymph nodes, postoperative complication rate and patients’ satisfaction with the cosmetic results, were analyzed.

**Results:**

The mean age of the patients was 42.3 ± 7.6 years in the ET group and 49.0 ± 10.8 years in the OT group (*P* = 0.003). The operation time was shorter in the COT group (112.3 ± 14min) than in the ET group (138.4 ± 36.9 min, *P*< 0.01). However, there were no significant differences in tumor size (0.5 ± 0.231vs. 0.41 ± 0.264cm, *P* = 0.116), number of retrieved lymph nodes (3.63 ± 2.1vs. 3.82 ± 3.28, *P* = 0.78) or postoperative hospital stay (3.35 ± 0.94vs. 3.17 ± 1.16 days, *P* = 0.457). Patients in the ET group experienced more pain than those in the COT group at 1 and 7 days after the operation as evaluated by a visual analog scale (*P* = 0.037, 0.026). Cosmetically, patients in the ET group were very satisfied with the operative procedure according to the questionnaire we used (1.43 ± 0.55vs. 3.21 ± 0.72, *P*< 0.001). The mean follow-up period was 54.3 months in the ET group and 47.4 months in the COT group, and each group exhibited one case of tumor recurrence detected at the other thyroid lobe within 2 years.

**Conclusions:**

Large series of prospective studies and long-term follow-up are needed, but the results of ET using the axillary approach for micropapillary thyroid carcinoma were not inferiortothose using COT, and it might be a safe and feasible procedure with good cosmetic results.

## Background

With the development of minimally invasive surgery, the use of endoscopic surgery for head and neck disease has also developed. Endoscopic neck surgery was first introduced by Gagner in 1996 [1], and Huscher performed the first endoscopic thyroidectomy in 1997 [2]. Thereafter, various endoscopic operative methods, including axillary, breast and anterior chest approaches,were introduced by many surgeons. Initially, endoscopic thyroid surgery was thought to be appropriate only for benign thyroid diseasesand contraindicated in thyroid cancer patients because a complete thyroidectomy could not be adequately performed via endoscopic means [3]. However, the prognosis of papillary thyroid carcinoma is relatively good.More females suffer from thyroid tumors than males. Because of its cosmetic advantages, ET has been carefully and gradually appliedto thyroid malignancies. As experience is accumulating and techniques are being developed, the indications for endoscopic thyroidectomy for thyroid malignancies can be expanded.The aim of this study was to compare endoscopic lobectomy and isthmectomy with ipsilateral central neck dissection by the axillary approach with the conventional open method in terms of the surgical outcomes and long-term courses for papillary thyroid microcarcinoma.

## Methods

From October 2002 to December 2008, a total of 41 patients with papillary thyroid microcarcinoma were operated on using the conventional open approach at Bucheon St. Mary’s Hospital. Additionally, in the same period, a series of 37 patients with papillary thyroid microcarcinoma underwent endoscopic thyroidectomy by the axillary approach at the same institution. Our indications for endoscopic thyroidectomy are as follows: tumor size not exceeding 1cm and no evidence of lymph node metastasis or local invasion on preoperative ultrasonography, computed tomography (CT) and physical examination.

All procedures were performed by one surgeon (K.Y.S.)andcarried out based strictly on patients’ individual decisions after giving informed consent concerning the surgical risks. Before the operations, we described the operative methods and differences between ET and COT, and each patient chose his or her own procedure. We analyzed the medical records of all patients retrospectively and evaluated surgical outcomes such as hospital day, operative time, complications, number of retrieved lymph nodes, tumor size and cosmetic results in the ET compared to the COT group.

The satisfaction with cosmetic results was analyzed quantitatively using a scoring systemthat ranged from 1 to 4(1, extremely; 2, fairly; 3,normal; 4: not at all) that was rated by patients at the outpatient clinic 6 months after the operation. The International Review Board of The Catholic University of Korea, Bucheon St. Mary’s Hospital, approved this study protocol.

### Surgical procedures

#### Endoscopic thyroidectomy

**Figure 1:**
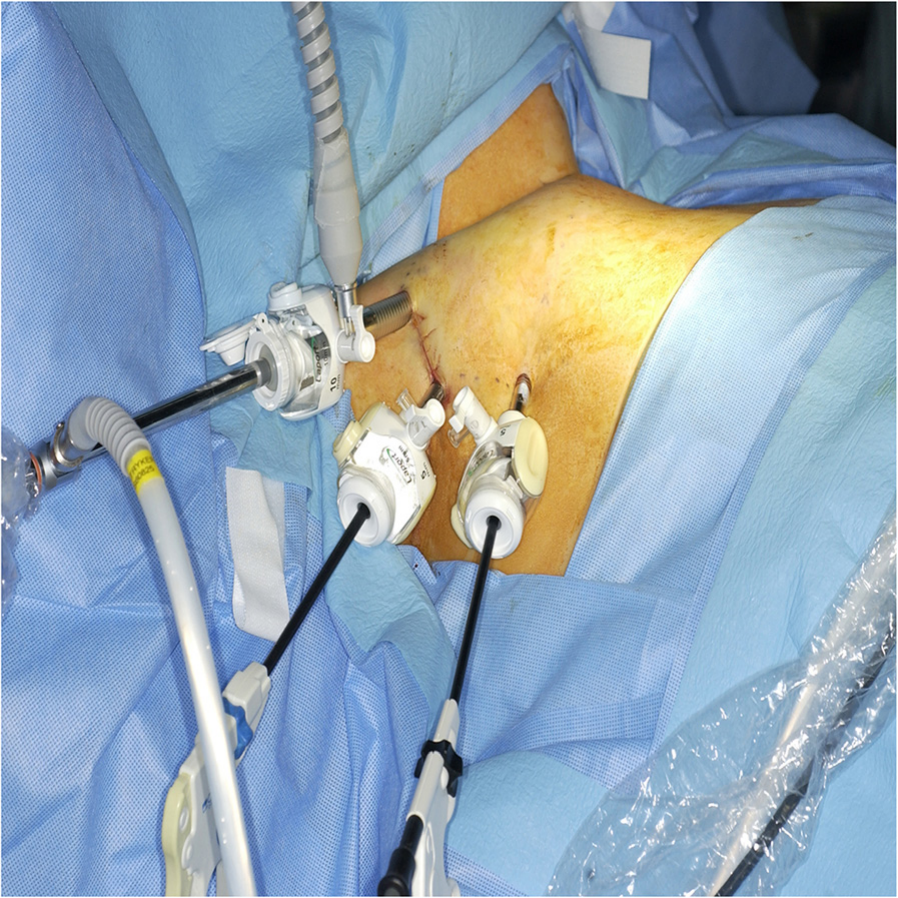
**Operative view of ipsilateral axillary approach endoscopic thyroidectomy.** Two trocars (5mm and 10mm) are inserted through axillary incisions. CO_2_ gas is insufflated via one 10-mm trocar, and the 10-mm rigid endoscope is inserted via this trocar.

Patients in the ET group underwent endoscopic lobectomy and isthmectomy with central lymph node dissection under general endotracheal anesthesia. The arm on the side of the ipsilateral nodule was raised, and the axilla was completely exposed. A diluted epinephrine solution (1:300,000) was injected into the subcutaneous layer of the anterior chest and cervical skin. A 30–40-mm skin incision was made in the axilla, and the lower layer of the platysma was exposed through the upper portion of the pectoralis major muscle. Trocars (one 10mm and one 5mm) were inserted into this incision, and a purse-string suture was placed to prevent gas leakage and slipping of the trocars out of the wound. Carbon dioxide (CO_2_) gas was then insufflated up to 6mmHg, and a 30° 10-mm rigid laparoscope was inserted into the 10-mm trocar. After adequate dissecting, one more 5-mm trocar was inserted near the 30-mm skin incision in the anterior axillary line (Figure 1). Endoscopic scissors were used for additional blunt and sharp dissection to enlarge the subplatysmal space.

After the sternocleidomastoid muscle and the strap muscle had been identified, the medial border of the sternocleidomastoid muscle was dissected from the strap muscle using a hook-typeharmonic scalpel. After the thyroid had been exposed, the upper pole of the thyroid was drawn downward, and superior thyroid vessels were identified and individually divided close to the thyroid to avoid injuring the external branch of the superior laryngeal nerve. During dissection, the superior parathyroid gland was identified and left intact.

**Figure 2:**
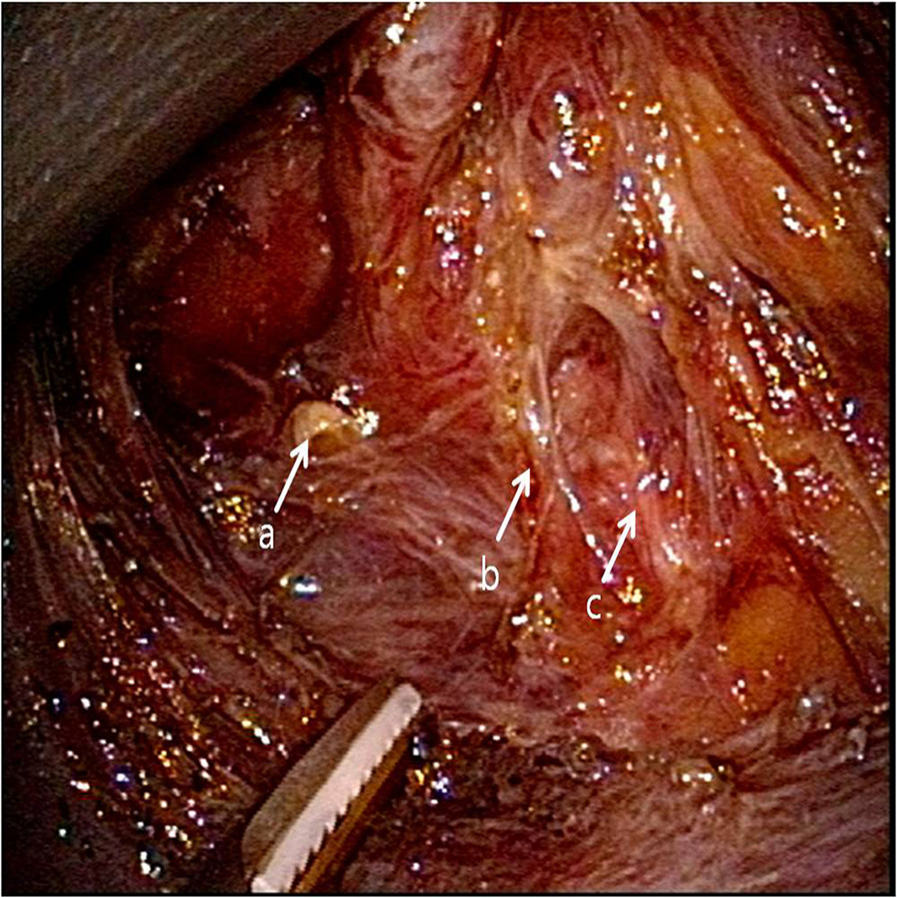
**Lateral view of the operative field during the operation.** The recurrent laryngeal nerve was separated carefully from the thyroid gland, and the inferior thyroid artery, which crossed the recurrent laryngeal nerve,was divided with a harmonic scalpel. **a **Superior parathyroid gland; **b** inferior thyroid artery; **c** recurrent laryngeal nerve.

Next, the thyroid was retracted medially, and the middle thyroid vein was identified and divided carefully. Careful dissection was performed to identify the inferior thyroid artery and the recurrent laryngeal nerve. The inferior thyroid artery was divided close to the thyroid, and the whole course of the recurrent laryngeal nerve was dissected (Figure 2). During the division of the inferior thyroid artery and dissection of the recurrent laryngeal nerve, the inferior parathyroid gland was identified and left intact. After the inferior thyroid vein and thyro-thymic ligament had been divided, the thyroid was retracted upward and medially. The lymph nodes of the central compartment were carefully dissected to avoid injury to the RLN. Finally, the thyroid was dissected from the trachea, and the isthmus was cut with a harmonic scalpel.

The specimen was extracted through the 30-mm skin incision. Before completing the operation, a 3-mm closed suction drain was placed under the platysma on the site of the most inferior 5-mm trocar in the axilla. The wounds were closed by tightly suturing the subcutaneous skin with 4–0 absorbable monofilament sutures using an atraumatic needle, followed by placement of Steri-Strips.

#### Open thyroidectomy

**Figure 3:**
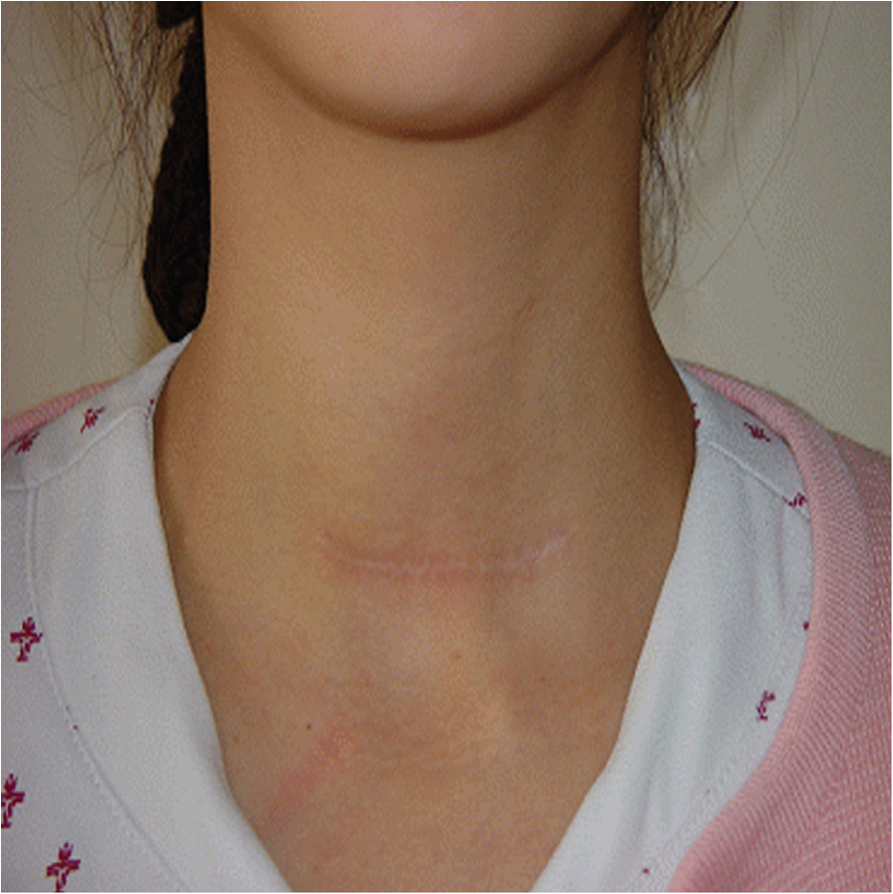
**Incision for conventional open thyroidectomy.** The length of the incision is 6.0 cm.

Patients in the COT group underwent conventional open thyroid lobectomy and isthmectomy with central lymph node dissection in the supine position with neck extension under general endotracheal anesthesia. A 6–8-cm transverse collar skin incision was made in the midline of the anterior neck 2cm above the sternal notch (Figure 3). The lower layer of the platysma was exposed, and subplatysmal flap dissection was performed from the sternal notch to the level of the hyoid bone superiorly. The midline of the strap muscles was divided vertically, and the thyroid gland was exposed. Dissection of the thyroid and division of the thyroid vessels and parenchyma were performed with electrocautery and ligation. In all cases, the recurrent laryngeal nerve and parathyroid glands were identified in the operative field. The wounds were closed with absorbable suture material by an interrupted subcutical suture method.

#### Postoperative care

There was no difference between the groups in postoperative care except for the closed drain in the ET group,which was usually removed on day 2 after the operation. Patients without any complications were usually discharged on day 2 after the operation.

#### Statistical analysis

The two groups were compared by Student’s *t*-test for continuous variables and expressed as the mean ± SD. Categorical variables were analyzed by chi-square test. *P*< 0.05 was considered significant. All statistical analyses were performed using the Statistical Package for the Social Sciences (SPSS), version 15.0, for Windows (SPSS, Inc., Chicago, IL, USA).

## Results

The clinicopathologic characteristics of 78 patients are summarized in Table 1. Subjects in the ET group were younger than those in the COT group. The mean age of the patients was 42.3 ± 7.6years in the ET group and 49.0 ± 10.8years in the COT group (*P* = 0.003). The pathological characteristics of both groups, such as the tumor size (0.5 ± 0.231vs. 0.41 ± 0.264cm, *P* = 0.116), number of patients who had thyroid capsular invasion (3 vs. 4, *P* = 0.799), number of extrathyroidal extensions (1 vs. 2, *P* = 0.618) and number of patients whose tumors showed multiplicity (3 vs. 5, 0.552), were not statistically different. The ET group had more patients with lymph node metastasis (5 vs. 1), but this didnot result in a final difference in the stage (according to the AJCC cancer staging, 7th edition) of both groups(*P* = 0.561). There were no statistically significant differences between the two groups in the number of retrieved lymph nodes (3.63 ± 2.1 vs. 3.82 ± 3.28, *P* = 0.78) and postoperative hospital stay (3.35 ± 0.94vs. 3.17 ± 1.16 days, *P* = 0.457). However, the operation time was shorter in the COT group (112.3 ± 14min) than in the ET group (138.4 ± 36.9 min, *P*< 0.01). In terms of postoperative pain, endoscopic thyroidectomy was more painful(VAS 1; *P* = 0.037, VAS 7; *P* = 0.026).

**Table 1:** Clinicopathologic characteristics of the patients in the two groups

	**ENDO**	**OPEN**	***P***
Characteristics			
Sex (male:female)	0:37	3:38	0.799
Age (years)	42.3 ± 7.6	49.0 ± 10.8	0.003
Tumor size (cm)	0.5 ± 0.231	0.41 ± 0.264	0.116
Capsule invasion (+/total)	3/37	4/41	0.799
Extrathyroid (+/total)	1/37	2/41	0.618
Multiplicity (+/total)	3/37	5/41	0.552
LN meta (+/total)	5/37	1/41	0.037
Stage			0.561
I/II/III/IV	34/0/3/0	39/0/2/0	
Number of retrieved lymph nodes	3.63 ± 2.1	3.82 ± 3.28	0.78
OP time (min)	138.4 ± 36.9	112.3 ± 14	<0.01
Hospital stay (days)	3.35 ± 094	3.17 ± 1.16	0.457
Complications	1	1	0.941
Bleeding	5	2	
Transient vocal cord palsy			
Paresthesia			
Postoperative pain			
VAS 1	4.7 ± 1.7	3.8 ± 1.9	0.037
VAS 7	2.6 ± 1.9	1.7 ± 1.8	0.026
Number of patients with neck discomfort			
POD 1 month	6	5	0.748
POD 3 months	3	2	0.664
Cosmesis	1.43 ± 0.55	3.21 ± 0.72	<0.001
Recurrence	1	1	0.667
Follow-up period (months)	54.3 ± 21.7	47.4 ± 13.6	0.1

*VAS 1* Visual analog scale 1 day after the operation.

*VAS 7* Visual analog scale 7 days after the operation.

The postoperative complications of both groups were checked in regard to recurrent laryngeal nerve palsy, postoperative bleeding, seroma, infection,etc. There was one postoperative complication in the ET group involving minor postoperative bleeding,which was treated with conservative care. One patient experienced transient vocal cord palsy in the COT group, but this resolved within 2 months without any treatment. After the hoarseness haddisappeared, we observed normal movement of the vocal cords by laryngoscope.

**Figure 4:**
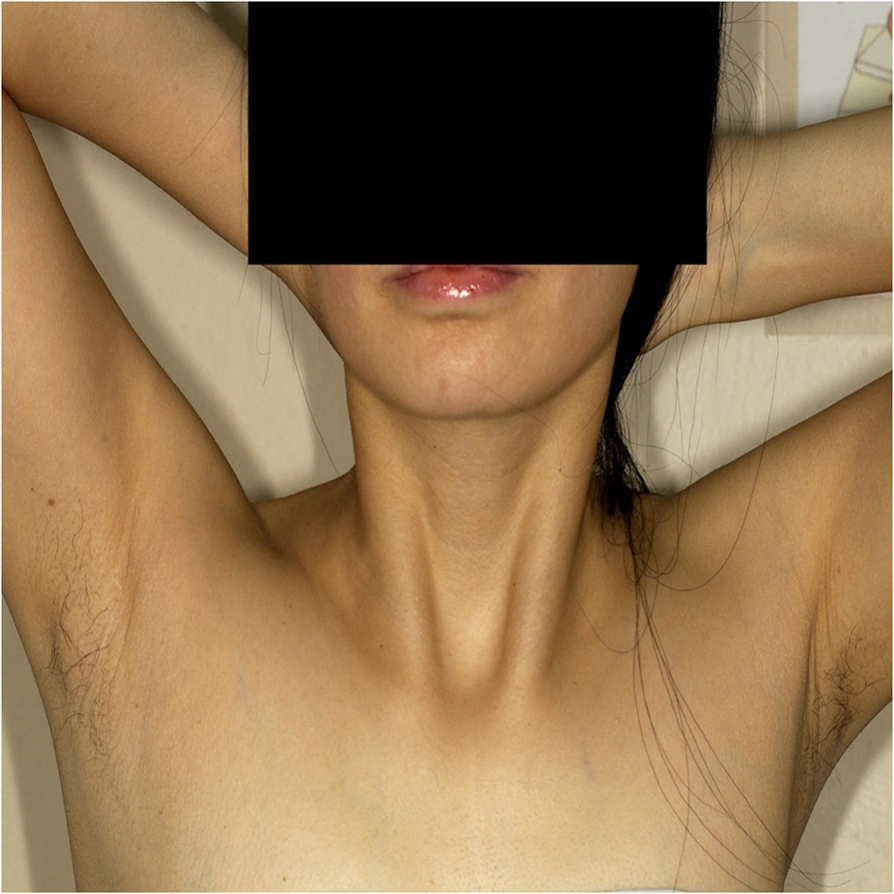
Macroscopic view of the neck and axilla 6 months after endoscopic thyroidectomy using the axillary approach.

The mean follow-up period was 54.3 ± 21.7months in the ET group and 47.4 ± 13.6months in the COT group. In the COT group, there was one case of tumor recurrence detected at the other thyroid lobe2 years later. Also, in the ET group, there was one case of recurrence in the other thyroid lobe 14 months later. The cosmetic result was evaluated using a scoring system (1, extremely; 2, fairly; 3,normal; 4, not at all) 6 months after operation. The postoperative cosmetic resultswere excellent in the ET group, and the small scar left along the skin crease in the axilla was completely covered by the patient’s arm in its natural position (Figure 4). In terms of cosmesis, we found that satisfaction with the cosmetic aspect of the operative procedure was higher in the ET group(1.43 ± 0.55vs. 3.21 ± 0.72, *P*< 0.001).

## Discussion

With the development of various surgical techniques and instruments, endoscopic surgery has been growing recently and attemptedin various surgical fields. Thyroid tumors usually occur in women, and the incidence rate is especially increased in young women. Patients are interested not only in the treatment of the disease, but also in the postoperative quality of life, especially concerning the operation scars. Therefore, surgeons have made efforts to resolve the scarring problem. With the improvements of laparoscopic instruments and increased surgeon experience, interest has focused ondeveloping less invasive instruments and surgical techniques to provide minimally invasive surgery also for the thyroid tumors [4]. Endoscopic neck surgery was attempted by Gagner in 1996 [1]. The first endoscopic thyroidectomy was performed by Huscher in 1997 [2]. Since then, various methods, including axillary, breast and anterior chest approaches, have been introduced by many surgeons [5-7].

Concerning the oncological safety, endoscopic thyroidectomy for malignant thyroid tumors is controversial. Initially, COT was the treatment of choice for malignant thyroid tumors [8]. However, papillary thyroid and follicular thyroid carcinomas have a very good prognosis, and the prognosis is not affected by whether central lymph node dissection is done [9]. Also, some reports state that if there is no lymph node metastasis, central lymph node dissection is not recommended because it may cause permanent hypoparathyroidism [9]. Other studies reported that complete thyroidectomy is not always necessary in patients with papillary thyroid microcarcinoma. Thyroid lobectomy alone may be a sufficient treatment for small (<1cm), low-risk, unifocal, intrathyroidal papillary carcinoma in the absence of prior head and neck irradiation or radiologically or clinically involved cervical nodal metastases [10]. The prospective study of endoscopic thyroidectomy for patients with papillary thyroid microcarcinoma was first done by Miccoli [11]. Recently, comparative studies of endoscopic thyroidectomy versus conventional open thyroidectomy have been reported [4,8,12,13]. These comparative studies reported that there was no significant difference regarding the technical safety between COT and ET, and theyfound that ET had many advantages, including better cosmetic results [12-17].

At first, we applied ET for benign thyroid tumors. As experience with this method has accumulated, the indications for ET have been expanded to some cases of thyroid malignancies. We used a unilateral axillary approach for endoscopic thyroidectomy and have applied it to malignant thyroid disease since 2002.

Many researchers have emphasized the definite indications and contraindications for endoscopic thyroidectomy. Kitano et al. reported the indicationsfor treating thyroid cancer with endoscopic surgery [18]. The indications for using endoscopic surgery for thyroid cancer are as follows: age <45 years, tumor size < 2cm, and no evidence of lymph node metastasis or local invasion. Miccoli et al. showed that the completeness of the results obtained with minimally invasive video-assisted thyroidectomy for thyroid cancers not exceeding 3.5cm in diameterwas similar to that obtained with open thyroidectomy [11,19]. Our indications for using endoscopic thyroidectomy for malignant thyroid disease in this study were as follows: tumor size not exceeding 1cm on preoperative ultrasonography; no evidence of lateral lymph node metastasis or local invasion on preoperative ultrasonography, computed tomography (CT), and physical examination; patient consent was obtained.

Many studies reported that there was no significant difference in the cervical lymph node dissection technique and surgical outcome between ET and COT [20-23]. It might enhance the surgical completeness of ET. Jeong et al. reported that the average number of retrieved lymph nodes was 5.05 ±2.94 (1–16) in ET, and there was no statistical difference compared to COT [4]. Along with the two other studies, the numbers were 5.38 and 3.5, respectively [16,24]. In our study, the numbers were 3.63 ± 2.1 in ET and 3.82 ± 3.28 in COT. The number was relatively small, but there was no statistical difference. Considering unilateral central lymph node dissection, it was not too small compared with the number of recent studies.

The incidence of transient recurrent laryngeal nerve palsy after conventional open thyroidectomy is reported to be 0% to 6% and that of permanent recurrent laryngeal nerve palsy less than 1% [25,26]. In our procedure, when the thyroid gland is freed or resected, we operated in close proximity to the thyroid capsule, and the RLN and parathyroid gland were magnified on a videoscope. Therefore, their preservation was easy. We didn’t encounter recurrent laryngeal nerve palsy in the ET group, although in the COT group, there was one patient whose symptom of transient vocal cord palsy spontaneously disappeared after 2 months.

Objective evaluation of cosmetic results is difficult at present. Cosmetic results are difficult to demonstrate without some bias because of the subjective judgment of the patients. In our study, cosmetic results were evaluated with a scoring system (1, extremely; 2, fairly; 3,normal; 4, not at all) 6 months after the operation, and all of the patients treated by the axillary approach described in this study were satisfied with their operative scars. Among the 37 patients who received endoscopic thyroidectomy, 17 were extremely satisfied with their cosmetic results. Some patients in the ET group complained of paresthesia, but this also disappeared 3 months after the operation.

Dhiman et al. reported disadvantages of endoscopic thyroidectomy with thyroid disease and thyroid cancer at 2008 [17]: using the equipment requirestwo to three assistants;there are a steep learning curve and longer duration of surgery;it isn’t applicable for thyroid glands> 20cc in size;costs are increasedbecause of equipment usage. The operation time for the COT group in our study was longer than thetimes reported in the literature [4,27,28]. We assumed that there were two reasons: First, we didn’t use drains to improve the patients’ cosmesis. Therefore, meticulous bleeding control and careful lymph node dissection required longer operation times. Secondly, most operations were performed without skilled assistants because of the lack of human resourcesin our department. Despite the longer operation time in the COT group than reported in the literature, the operation time in the ET group in our study was even longer than that inthe COT group. Recently, we decreased the operation time in the ET group to about 120 min (data not shown). We investigated short-term postoperative pain to evaluate the invasiveness of endoscopic thyroidectomy using the axillary approach. Both groups showed no significant difference in neck discomfort 3 months after the operation, but endoscopic thyroidectomy definitely was more painful than COT in the short term. We assumed that this disadvantage was due to wider dissection in the ET group than in the COT group. To minimize this disadvantage, other methods such as a single-port surgery will be helpful.

In our study, one case of recurrence occurred in each group, and we performed completion thyroidectomy. In terms of multiplicity, there were three cases in the ET group and five cases in the COT group. All were closely observed with the patient’s consent and no additional operations.

As we described, endoscopic thyroidectomy was safe and effective for treating papillary thyroid microcarcinoma. According to the literature, endoscopic thyroidectomy is an acceptable method for treating T2 tumors, T3 tumors and lateral neck lymph node dissection [23,28,29]. According to the results of this study, we are going to broaden our indications.

## Conclusion

Endoscopic thyroid lobectomy with central lymph node dissection using the axillary approach is a safe, minimally invasive procedure that produces outcomes similar to those of conventional open thyroidectomy when considering short-term adverse events.It has the advantage of achieving better cosmetic results than open thyroidectomy. Although larger randomized controlled trials with a longer follow-up are needed, endoscopic thyroidectomy is an effective alternative for selected patients with papillary thyroid microcarcinoma compared to conventional open thyroidectomy.

## Competing interests

The authors declare that they have no competing interest.

## Authors’ contribution

H Lee drafted the manuscript. J lee was responsible for analysis and interpretation of data. KY Sung supervised the research, the revision of the manuscript and was the corresponding author. All authors read and approved the final manuscript.

## References

[B1] Gagner M Endoscopic subtotal parathyroidectomy in patients with primary hyperparathyroidism Br J Surg 1996 83 875 10.1002/bjs.1800830656 8696772

[B2] Hüscher CS Chiodini S Napolitano C Recher A Endoscopic right thyroid lobectomy Surg Endosc 1997 11 877 10.1007/s004649900476 9266657

[B3] Duh Q-Y Presidential address: Minimally invasive endocrine surgery–standard of treatment or hype? Surgery 2003 134 849 857 10.1016/S0039-6060(03)00405-7 14668714

[B4] Jeong JJ Kang S-W Yun J-S Sung TY Lee SC Lee YS Nam K-H Chang HS Chung WY Park CS Comparative study of endoscopic thyroidectomy versus conventional open thyroidectomy in papillary thyroid microcarcinoma (PTMC) patients J Surg Oncol 2009 100 477 480 10.1002/jso.21367 19653245

[B5] Ohgami M Ishii S Arisawa Y Ohmori T Noga K Furukawa T Kitajima M Scarless endoscopic thyroidectomy: breast approach for better cosmesis Surg Laparosc Endosc Percutan Tech 2000 10 1 4 10872517

[B6] Ikeda Y Takami H Niimi M Kan S Sasaki Y Takayama J Endoscopic thyroidectomy by the axillary approach Surg Endosc 2001 15 1362 1364 10.1007/s004640080139 11727158

[B7] Shimazu K Shiba E Tamaki Y Takiguchi S Taniguchi E Ohashi S Noguchi S Endoscopic thyroid surgery through the axillo-bilateral-breast approach Surg Laparosc Endosc Percutan Tech 2003 13 196 201 10.1097/00129689-200306000-00011 12819505

[B8] Sgourakis G Sotiropoulos GC Neuhäuser M Musholt TJ Karaliotas C Lang H Comparison between minimally invasive video-assisted thyroidectomy and conventional thyroidectomy: is there any evidence-based information? Thyroid 2008 18 721 727 10.1089/thy.2008.0028 18631000

[B9] Shaha A Treatment of thyroid cancer based on risk groups J Surg Oncol 2006 94 683 691 10.1002/jso.20697 17131422

[B10] Cooper DS Doherty GM Haugen BR Hauger BR Kloos RT Lee SL Mandel SJ Mazzaferri EL McIver B Pacini F Schlumberger M Sherman SI Steward DL Tuttle RM Revised American Thyroid Association management guidelines for patients with thyroid nodules and differentiated thyroid cancer Thyroid 2009 19 1167 1214 10.1089/thy.2009.0110 19860577

[B11] Miccoli P Elisei R Materazzi G Capezzone M Galleri D Pacini F Berti P Pinchera A Minimally invasive video-assisted thyroidectomy for papillary carcinoma: a prospective study of its completeness Surgery 2002 132 1070 1073 discussion 1073–1074 10.1067/msy.2002.128694 12490857

[B12] Yamamoto M Sasaki A Asahi H Shimada Y Saito K Endoscopic versus conventional open thyroid lobectomy for benign thyroid nodules: a prospective study Surg Laparosc Endosc Percutan Tech 2002 12 426 429 10.1097/00129689-200212000-00007 12496549

[B13] Chung YS Choe J-H Kang K-H Kim SW Chung K-W Park KS Han W Noh D-Y Oh SK Youn Y-K Endoscopic thyroidectomy for thyroid malignancies: comparison with conventional open thyroidectomy World J Surg 2007 31 2302 2306 discussion 2307–2308 10.1007/s00268-007-9117-0 17566819

[B14] Ikeda Y Takami H Sasaki Y Takayama J Niimi M Kan S Comparative study of thyroidectomies Endoscopic surgery versus conventional open surgery Surg Endosc 2002 16 1741 1745 10.1007/s00464-002-8830-x 12140635

[B15] Lombardi CP Raffaelli M Princi P Lulli P Rossi ED Fadda G Bellantone R Safety of video-assisted thyroidectomy versus conventional surgery Head Neck 2005 27 58 64 10.1002/hed.20118 15459914

[B16] Yoo H Chae BJ Park HS Kim KH Kim SH Song BJ Jung SS Bae JS Comparison of surgical outcomes between endoscopic and robotic thyroidectomy J Surg Oncol 2012 105 705 708 10.1002/jso.22106 21953060

[B17] Dhiman SV Inabnet WB Minimally invasive surgery for thyroid diseases and thyroid cancer J Surg Oncol 2008 97 665 668 10.1002/jso.21019 18493913

[B18] Kitano H Fujimura M Kinoshita T Kataoka H Hirano M Kitajima K Endoscopic thyroid resection using cutaneous elevation in lieu of insufflation Surg Endosc 2002 16 88 91 10.1007/s004640080197 11961612

[B19] Miccoli P Berti P Raffaelli M Materazzi G Baldacci S Rossi G Comparison between minimally invasive video-assisted thyroidectomy and conventional thyroidectomy: a prospective randomized study Surgery 2001 130 1039 1043 10.1067/msy.2001.118264 11742335

[B20] Ikeda Y Takami H Sasaki Y Takayama J Kan S Niimi M Minimally invasive video-assisted thyroidectomy and lymphadenectomy for micropapillary carcinoma of the thyroid J Surg Oncol 2002 80 218 221 10.1002/jso.10128 12210037

[B21] Shimizu K Kitagawa W Akasu H Tanaka S Endoscopic hemithyroidectomy and prophylactic lymph node dissection for micropapillary carcinoma of the thyroid by using a totally gasless anterior neck skin lifting method J Surg Oncol 2001 77 217 220 10.1002/jso.1098 11455561

[B22] Kitagawa W Shimizu K Akasu H Tanaka S Endoscopic neck surgery with lymph node dissection for papillary carcinoma of the thyroid using a totally gasless anterior neck skin lifting method J Am Coll Surg 2003 196 990 994 10.1016/S1072-7515(03)00130-3 12788440

[B23] Lombardi CP Raffaelli M Princi P De Crea C Bellantone R Minimally invasive video-assisted functional lateral neck dissection for metastatic papillary thyroid carcinoma Am J Surg 2007 193 114 118 10.1016/j.amjsurg.2006.02.024 17188101

[B24] Hur SM Kim SH Lee SK Kim WW Choe J-H Lee JE Kim J-H Nam S-J Yang J-H Kim JS New endoscopic thyroidectomy with the bilateral areolar approach: a comparison with the bilateral axillo-breast approach Surg Laparosc Endosc Percutan Tech 2011 21 e219 e224 10.1097/SLE.0b013e3182239989 22002279

[B25] Gourin CG Johson JT Randolph GW Postoperative complication Surgery of the Thyroid and Parathyroid Glands 2002 1 Philadelphia: Saunders 433 443

[B26] Randolph GW Kobler JB Wilkins J Recurrent laryngeal nerve identification and assessment during thyroid surgery: laryngeal palpation World J Surg 2004 28 755 760 10.1007/s00268-004-7348-x 15457354

[B27] Hong HJ Kim WS Koh YW Lee SY Shin YS Koo YC Park YA Choi EC Endoscopic thyroidectomy via axillo-breast approach without gas insufflations for benign thyroid nodules and micro papillary carcinomas: Preliminary results Yonsei Med J 2011 52 643 654 10.3349/ymj.2011.52.4.643 21623608 PMC3104441

[B28] Lombardi CP Raffaeli M De Crea C Princi P Castaldi P Salvatori M Bellantone R Report on 8 years of experienced with video-assisted thyroidectomy for papillary thyroid carcinoma Surgery 2007 142 944 951 10.1016/j.surg.2007.09.022 18063080

[B29] Lombardi CP Raffaeli M Princi P De Crea C Bellantone R Minimally invasive video-assisted functional lateral neck dissection for metastatic papillary thyroid carcinoma Am J Surg 2007 193 114 118 10.1016/j.amjsurg.2006.02.024 17188101

